# Mignot and Yanagisawa win Lasker prize for discovery of the role of orexins in wake, sleep, and narcolepsy

**DOI:** 10.1172/JCI211621

**Published:** 2026-09-09

**Authors:** Clifford B. Saper

**Affiliations:** Department of Neurology, Division of Sleep Medicine, and Program in Neuroscience, Beth Israel Deaconess Medical Center and Harvard Medical School, Boston, Massachusetts, USA.

The Albert Lasker Basic Medical Research Award for 2026 has been awarded to Dr. Emmanuel Mignot and Dr. Masashi Yanagisawa for the identification of the role of orexins (also known as hypocretins) in the regulation of sleep and wakefulness and that deficiency of orexin signaling is the cause of the sleep disorder, type 1 narcolepsy. Their discoveries were the culmination of complementary lines of research that led to the understanding of both brain functions and disorders that have long puzzled scientists and physicians and to the development of new drugs that improve patients’ lives.

## New insights into narcolepsy

Orexins/hypocretins were named by two independent lab groups who discovered them in the late 1990s. Luis de Lecea and Greg Sutcliffe used a screen for peptides that were most highly expressed in the hypothalamus to find the mRNA for the peptides they named hypocretins (1). While they were doing this work, Masashi Yanagisawa and Takeshi Sakurai screened for ligands for orphan G protein–coupled receptors and identified the orexin peptides in brain, reporting their definitive structure, gene, and receptors (2). Their reports were published a month apart, and only after reading both papers did scientists realize that the two groups had discovered the same peptides.

The name hypocretins reflected a homology of the peptides with secretin, while the term orexins was chosen because the only neurons in the brain that express the peptides were in the lateral hypothalamus, which had previously been associated with regulation of feeding. Injection of the orexins into the lateral ventricle indeed caused feeding when this was done in the morning when mice are generally asleep (2). However, the injections at other times of day did not cause feeding (3). In an effort to solve this paradox, Yanagisawa’s group generated orexin-null mice (4). Although the mice had only subtle differences in feeding and body weight, other experiments showed that orexin neurons are more active during wakefulness than during sleep. Infrared video imaging of the mice soon showed that during the night, when mice usually are awake, they would have brief attacks of immobility or would suddenly fall asleep, particularly while interacting with their cagemates. These symptoms, sleep attacks and immobility due to sudden loss of muscle tone while awake, are hallmarks of narcolepsy.

Again, by remarkable coincidence, Emmanuel Mignot and his colleagues were studying the basis of inherited canine narcolepsy. By painstaking genetic analysis, they deduced that inherited narcolepsy was caused by a mutation in the hypocretin 2 receptor gene (also known as *OX2R*) (5). In August 1999, both groups published these results and described their findings in *Cell*, two weeks apart, and at a stroke the mystery of the origin of narcolepsy was solved; it could be caused by the loss of a single neurotransmitter in a single group of neurons in the hypothalamus, or by its type 2 receptor.

**Figure 1:**
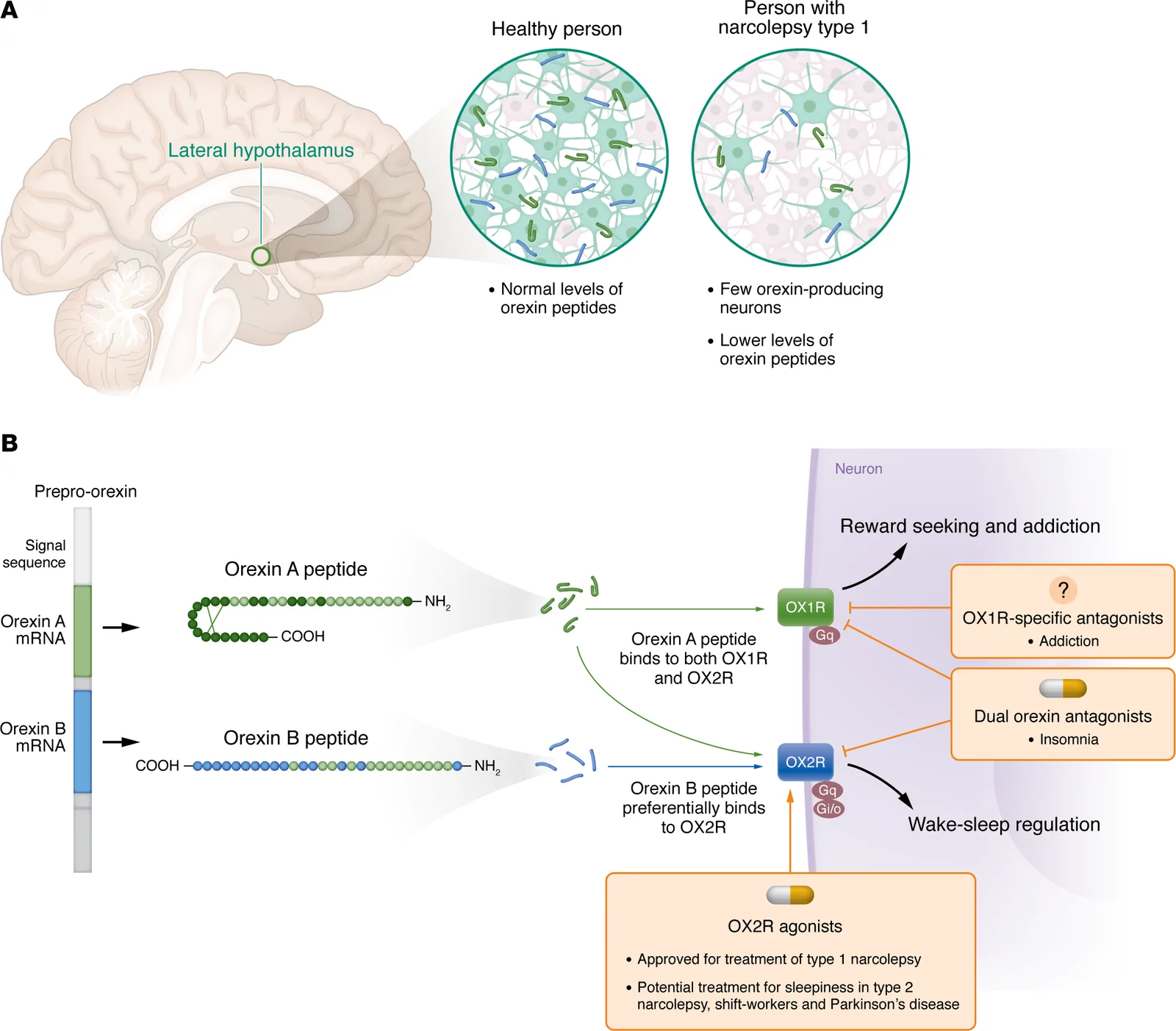
Molecular pharmacology of orexin action. (**A**) A schematic drawing showing the location of the orexin neurons in the lateral hypothalamus and the loss of orexin neurons in a patient with type 1 narcolepsy. (**B**) Shows a schematic view of the orexin gene, which contains the sequences for both the orexin A and orexin B peptides. Orexin A acts on both receptors, OX1R and OX2R, but orexin B acts mainly on OX2R. OX2R has been associated mainly with wake-sleep regulation and OX1R with reward seeking and addiction behavior. Dual orexin receptor antagonists are being used clinically to treat insomnia, but specific OX1R antagonists are under development to treat addiction and OX2R agonists are being tested for treating type 1 narcolepsy and may be useful in other disorders of increased daytime sleepiness. Adapted with permission from the *New England Journal of Medicine* (12).

Further studies showed that human narcolepsy is only rarely caused by mutations in orexin receptors, but it is mainly due to loss of the orexin neurons in the hypothalamus (Figure 1) (6, 7). The availability of a spinal fluid assay for orexin soon led to the discovery that patients with narcolepsy with cataplexy almost invariably had low levels of orexin in their spinal fluid, while patients with sleep attacks but without cataplexy tended to have much higher orexin levels, separating the two groups into type 1 and type 2 narcolepsy, respectively (8). Type 1 narcolepsy typically starts in the second or third decade of life, prime time for the onset of various autoimmune disorders. Spikes in the incidence of type 1 narcolepsy after epidemics of H1N1 influenza in China and after administering an H1N1 vaccine in Scandinavia supported an autoimmune origin (9, 10).

## From molecular target to therapeutic development

Work subsequently proceeded on development of drugs to manipulate orexin transmission. Orexin neurons innervate a wide range of brain targets, from the cerebral cortex to many of the monoamine cell groups in the brainstem; however, the distribution of the type 1 and type 2 orexin receptors (OX1Rs and OX2Rs) is distinct (11). OX2Rs are most prominent in the deep layers of the cerebral cortex and on a variety of wake-promoting cell groups in the basal forebrain, thalamus, hypothalamus, and brainstem. OX1Rs are more prominent in certain hypothalamic nuclei as well as in brainstem cholinergic, dopaminergic, and noradrenergic cell groups. Evidence from behavioral studies indicates that OX2Rs may be more important for maintaining a wakeful state, whereas OX1Rs may play a role in addiction (12).

In general, it is usually easier to develop drugs that interfere with binding at peptidergic G protein–coupled receptors than to make ones that act as agonists, and so it has been with the orexin receptors. The first drugs to reach approval for use in humans were OX1R/OX2R dual antagonists, such as suvorexant, daridorexant, and lemborexant, which are used to promote sleep in patients with insomnia (13). These drugs are particularly useful in patients who have intolerable side effects with other sedative/hypnotic drugs (such as GABA receptor enhancers, including benzodiazepines and related drugs that cause ataxia and falls; and anticholinergic or antihistaminergic drugs that may cause confusion and have cardiovascular or urinary side effects). Current work is attempting to develop selective OX1R antagonists, which can potentially be used to treat addiction without causing daytime sleepiness (14).

The development of orexin receptor agonists has been slower. The first generation of selective OX2R agonists such as danavorexton were only available for parenteral use, but they showed dramatic improvement in daytime sleepiness in patients with narcolepsy (15). Recently, the orally available OX2R agonist oveporexton was approved by the FDA for use in patients with type 1 narcolepsy (16). It is likely that OX2R agonists will eventually find more widespread use to treat daytime sleepiness due to type 2 narcolepsy, other hypersomnias, and shift-work and non-24 disorders that cause daytime sleepiness (17). In addition, the loss of orexin neurons in the hypothalamus in Parkinson’s disease suggests that OX2R agonists may be useful in preventing daytime sleepiness in those patients (18).

## Lessons from this discovery

The arc of this story, from the discovery of the orexin peptides in 1998 to the development of drugs addressing the orexin receptors that may be used to treat a variety of disabling human conditions, is a case study in the best of what modern science can provide to the world. It began with discovering the basis of a puzzling disease, contributed to our larger understanding of the regulation of sleep and wakefulness, and ultimately led to the development of new classes of drugs that address real human needs. However, its message for the value of basic scientific research is particularly piquant in these times. The initial work that led to the discovery of the orexin peptides and their roles was entirely untargeted, open-ended discovery science. This type of science, which is currently under threat, has repeatedly led to enormously important avenues for treating patients, from the work of previous Lasker Award winners, which led to advances such as the development of mRNA vaccines and GLP-1 drugs, to the current award. The second message to take away is that science is at its best when it is done by a global community with minimal restrictions on communication and collaboration. Mignot came from France, de Lecea from Spain, and Yanagisawa and Sakurai from Japan. All of these investigators were working in the US when they made their major discoveries. Policies that put a chill on international collaboration and prevent brilliant young investigators from pursuing their careers in the US are likely to be costly to the US and the world, and they will deprive unknown millions of people of drugs and other treatments that could improve or even save their lives.

Mignot and Yanagisawa are to be congratulated in producing the type of “touchdown” in the game of life that every basic scientist dreams about. It is incumbent on the rest of us to make sure that the conditions that led to this and other momentous discoveries are preserved for today’s young people and future generations.

## Conflict of interest

The author has declared that no conflict of interest exists.

## Version 1 09/09/2026

Electronic publication

## Version 2 09/15/2026

Electronic publication
